# PI3K-regulated Glycine N-methyltransferase is required for the development of prostate cancer

**DOI:** 10.1038/s41389-022-00382-x

**Published:** 2022-02-23

**Authors:** Amaia Zabala-Letona, Amaia Arruabarrena-Aristorena, Sonia Fernandez-Ruiz, Cristina Viera, Onintza Carlevaris, Amaia Ercilla, Isabel Mendizabal, Teresa Martin, Alice Macchia, Laura Camacho, Mikel Pujana-Vaquerizo, Pilar Sanchez-Mosquera, Verónica Torrano, Natalia Martin-Martin, Patricia Zuniga-Garcia, Mireia Castillo-Martin, Aitziber Ugalde-Olano, Ana Loizaga-Iriarte, Miguel Unda, Jose M. Mato, Edurne Berra, Maria L. Martinez-Chantar, Arkaitz Carracedo

**Affiliations:** 1 Center for Cooperative Research in Biosciences (CIC bioGUNE), Basque Research and Technology Alliance (BRTA), Bizkaia Technology Park, Building 801A, 48160 Derio, Spain; 2 CIBERONC, Madrid, Spain; 3 Ikerbasque, Basque Foundation for Science, Bilbao, Spain; 4 Traslational prostate cancer Research lab, CIC bioGUNE-Basurto, Biocruces Bizkaia Health Research Institute, Derio, Spain; 5 Biochemistry and Molecular Biology Department, University of the Basque Country (UPV/EHU), Bilbao, Spain; 6 Department of Pathology, Fundação Champalimaud, Lisboa, Portugal; 7 Department of Pathology, Basurto University Hospital, 48013 Bilbao, Spain; 8 Department of Urology, Basurto University Hospital, 48013 Bilbao, Spain; 9 Centro de Investigación Biomédica en Red de Enfermedades Hepáticas y Digestivas (CIBERehd), Madrid, Spain

**Keywords:** Prostate cancer, Cancer metabolism

## Abstract

Glycine N-Methyltransferase (GNMT) is a metabolic enzyme that integrates metabolism and epigenetic regulation. The product of GNMT, sarcosine, has been proposed as a prostate cancer biomarker. This enzyme is predominantly expressed in the liver, brain, pancreas, and prostate tissue, where it exhibits distinct regulation. Whereas genetic alterations in *GNMT* have been associated to prostate cancer risk, its causal contribution to the development of this disease is limited to cell line-based studies and correlative human analyses. Here we integrate human studies, genetic mouse modeling, and cellular systems to characterize the regulation and function of GNMT in prostate cancer. We report that this enzyme is repressed upon activation of the oncogenic Phosphoinositide-3-kinase (PI3K) pathway, which adds complexity to its reported dependency on androgen signaling. Importantly, we demonstrate that expression of GNMT is required for the onset of invasive prostate cancer in a genetic mouse model. Altogether, our results provide further support of the heavy oncogenic signal-dependent regulation of GNMT in prostate cancer.

## Introduction

Prostate cancer (PCa) is among the most frequent cancer types in men and accounts for a significant mortality by the disease [1]. The clinical management of this tumor type has profoundly changed with the implementation of prostate specific antigen (PSA)-based screening, minimally invasive robotic surgery, and new-generation androgen synthesis and signaling inhibitors [2, 3]. However, the molecular understanding of the disease has not yet been fully exploited for cancer stratification and therapy, as compared to other tumor types.

Prostate tumor cells rely on the abnormal activation of the PI3K pathway [1]. Large-scale genome aberration studies corroborated the initial observations related to Phosphatase And Tensin Homolog (*PTEN*) copy number alterations in prostate tumors [4–6]. Furthermore, mouse modeling experiments demonstrated that the prostate is exquisitely sensitive to *Pten* levels, and perturbation of this gene alone can unleash a cancerous phenotype [7–9]. Many effector pathways have been accounted for the action of the PI3K in prostate cancer [10]. Among these, the reprogramming of cancer metabolism has gained attention in recent years. In this regard, we have reported that a *Pten*-deletion based PCa mouse model recapitulates metabolic features of human PCa, with a predominant increase in the production of polyamines that are required for the proliferation of prostate tumor cells [9]. Polyamine metabolism stems from the catabolism of methionine and arginine [11]. Methionine is transformed into S-adenosylmethionine (SAMe) by MAT enzymes, and this metabolite can follow two different fates [11]. On the one hand, demethylation of SAMe by methyltransferases leads to the production of S-adenosylhomocysteine (SAH), which can enter the trans-sulfuration or remethylation pathway. The demethylation of SAMe is carried out by a variety of enzymes, including the Glycine N-methyltransferase (GNMT) [12]. On the other hand, SAMe can be decarboxylated by Adenosylmethionine Decarboxylase 1 (AMD1) to produce dcSAMe [11]. The control of AMD1 levels and activity by mTOR complex 1 (mTORC1) provides a feasible explanation for the elevated production of polyamines upon *PTEN* deletion [9].

GNMT is a metabolic enzyme predominantly expressed in the liver, pancreas, brain, and prostate tissue [12]. It catalyzes the transfer of a methyl group from SAMe to Glycine, resulting in the production of SAH and sarcosine. This enzyme is a well-accepted tumor suppressor in the liver [12]. Deletion of *Gnmt* in the mouse results in steatosis, steatohepatitis, and development of hepatocarcinoma late in life [13]. In contrast, various reports suggest that this enzyme favors various oncogenic features in PCa cells [14, 15]. GNMT overexpression in cell lines, or the supplementation with its product sarcosine, increases several oncogenic characteristics according to various reports [14–18]. In patients, there are conflicting results, posing GNMT as an enzyme overexpressed [14] and downregulated [19] in PCa. This discrepancy could be due to the distinct nature of the patient cohorts analyzed, or to the underlying molecular regulation of this enzyme by cancer-relevant factors such as MYC, ERG, or androgen receptor [15, 20–22]. Moreover, small nucleotide polymorphisms in *GNMT* locus have been associated to PCa risk [19, 23], although their functional impact on the enzyme remains obscure. Importantly, there is little evidence of the contribution of GNMT to PCa pathogenesis using immunocompetent genetic mouse models that are faithful to the human disease to date.

In this study, we provide a formal demonstration of the essential nature of GNMT expression for the development of PCa, and uncover a new regulatory mode of this enzyme downstream the oncogenic PTEN-PI3K pathway.

## Methods

### Patient recruitment

Human prostate tissue specimens were obtained from the Basque Biobank for research (BIOEF, Basurto University hospital). According to the Declaration of Helsinki principles, all participants in the study provided written informed consent to clinical investigations using an approved consent form and with evaluation and approval from the corresponding ethics committee (CEIC-E 11-12, 14-14, and 19-20). All data were double-codified to protect the confidentiality of individual participants. Tissue was accrued and processed following the protocol reported [24]. The clinical-pathological characteristics of the cases included in this study are presented in Supplementary Table 1.

### Animals

All mouse experiments were carried out following the ethical guidelines established by the Biosafety and Animal Welfare Committee at CIC bioGUNE. The procedures employed were carried out following the recommendations from AAALAC. GEMM experiments were carried out in a mixed background as reported [25]. The *Pten*^lox^ conditional knockout allele has been described elsewhere [7]. The prostate epithelium-specific deletion was effected by the Pb-Cre4 [7]. Orchiectomy was performed in 6 months *Pten*^*pc−/−*^ mice, and 6 days later prostate lobules were collected. Mice were fasted for 6 h prior to tissue harvest in order to prevent metabolic alterations due to immediate food intake. The *Gnmt* knockout mouse model was previously generated and characterized [13]. These mice are whole body mutants and reportedly develop steatosis and hepatocarcinoma, in line with the predominant expression of *Gnmt* in this organ. No randomization was used when comparing genotypes. Castration experiments were performed as reported [26].

### Reagents

Cell lines were purchased from Leibniz-Institut DSMZ—Deutsche Sammlung von Mikroorganismen und Zellkulturen GmbH (DSMZ) and tested negative for mycoplasma. Low passage stock cell lines were tested for cell identity validation. Rapamycin (prepared in DMSO, final concentration 20 nM), MK-2206 (prepared in DMSO, final concentration 500 nM), BKM120 (prepared in DMSO, final concentration 5 µM), MDV-3100 (prepared in DMSO, final concentration 10 µM), I-BET762 (prepared in DMSO, final concentration 1 uM), and DHT (4,5α-Dihydrotestosterone, dissolved in ethanol, final concentration 10 nM) were purchased from LC laboratories (Rapamycin), ShelleckChem (BKM120, MK-2206), SCBT (MDV-3100), CAYMAN (I-BET762), and Sigma-Aldrich (DHT). Doxycycline was purchased from Sigma and used at 500 ng/mL for over-expression of YFP-PTEN.

shRNAs against *GNMT* (TRCN0000000326: sh1; TRCN0000000329: sh4) and *FOXO1* (TRCN0000039582) were purchased from Sigma and control shRNA sequence is included (CCGGCAACAAGATGAAGAGCACCAACTCGAGTTGGTGCTCTTCATCTTGTTG). YFP-PTEN lentiviral constructs were described in [27].

### Cellular and molecular assays

Cell number quantification was done with crystal violet [28]. Foci assays were performed seeding 500 cells per well (6-well plate) and staining and counting them by crystal violet. Lentiviral and retroviral transductions were performed as previously described [25]. Western blot was performed as previously described [29]. The following antibodies were used for Western blotting: rabbit pAnti-RpS6^S240/244^, anti-RpS6, anti-HSP90, anti-PTEN (138G6), anti-AKT^S473^, anti-AKT (all dilution 1:1000) and anti-cleaved PARP (D64E10)(dilution 1:1000) were all from Cell Signalling. RNA was extracted using NucleoSpin® RNA isolation kit from Macherey-Nagel (ref: 740955.240 C). 1 μg of total RNA was used for cDNA synthesis using qScript cDNA Supermix from Quanta (ref. 95048). Quantitative Real Time PCR (q-RTPCR) was performed as previously described [29]. Applied biosystems TaqMan probes: *Gnmt/GNMT* (Mm00494688_m1, Hs00219089_m1), *FOXO1* (Hs00231106_m1) *GAPDH/Gapdh* (Hs02758991_g1/Mm99999915_g1), and *B-ACTIN* (Hs 99999903 m1).

### Immunohistochemical analysis

After euthanasia, histochemical analysis of a hematoxylin and eosin (H&E)–stained section from formalin-fixed paraffin-embedded prostate tissues was performed as previously described [25]. Scoring system performed by pathologist was based on: Normal (0); Increased cellularity (+1); Low-grade prostate intraepithelial neoplasia (LGPIN) (+2); High-grade PIN (HGPIN) (+3); Cancer (+4).

### Metabolomic analysis

For in vitro metabolomic analysis, growing cells were washed with ammonium carbonate pH 7.4 and snap-frozen in liquid nitrogen. Metabolites were extracted from cells or tissues with cold 80/20 (v/v) methanol/water. Samples were then dried and stored at −80 °C until MS analysis. Quantitative LC/MS was conducted as previously described [30] at AGIOS Pharmaceuticals. A Thermo Accela 1250 pump delivered a gradient of 0.025% heptafluorobutyric acid, 0.1% formic acid in the water, and acetonitrile at 400 µL/min. The stationary phase was an Atlantis T3, 3 µm, 2.1 x 150 mm column. A QExactive Mass Spectrometer was used at 70.000 resolving power to acquire data in full-scan mode. Data analysis was conducted in MAVEN [31] and Spotfire. Peak areas derived from stable isotope labelling experiments have been corrected for naturally occurring isotope abundance.

### ENCODE visualization

FOXO1 ChIP-Seq data of two replicates (“bed narrowPeak conservative IDR thresholded peaks” files from experiment ENCSR321OAA) were downloaded from https://www.encodeproject.org/experiments/ENCSR321OAA/. ENCODE Candidate Cis-Regulatory Elements (cCREs) EH38E2468125, EH38E2468126 and EH38E2468127 were represented in the Figure. Gviz R package [32] was used for visualization.

### Gene expression analyses from patient datasets

Data were obtained from the public repository GEO [33], and cBioPortal [34, 35]. All the datasets used, except Varambally [36], were downloaded as normalized data, which was corroborated by performing quality controls by means of boxplots and principal component analysis (PCA). For Varambally, a normalization by quartiles was applied [37] and a log2 transformation was performed. In addition, probes from this dataset that could lead to error due to cross-hybridizations were removed.

For gene expression comparisons among groups Student *T* test or ANOVA was performed as indicated. For pairwise correlations between the expression of selected genes, primary tumor samples were selected, and the Pearson correlation coefficient and *p* value was calculated as indicated in the graphs.

### Statistical analysis

No statistics were applied to determine the sample size. Mice for in vivo analyses were selected based on genotype, without randomization required. For castration experiments, mice of the selected genotype were randomly assigned to the experimental groups. The investigators were not blinded to allocation during experiments and outcome assessment. Data analysed by parametric tests are represented by the mean ± s.e.m. of pooled experiments and for non-parametric tests median with interquartile range is depicted, unless otherwise stated. n values represent the number of independent experiments performed or the number of individual mice or patient specimens. For each independent in vitro experiment, at least two technical replicates were used and a minimum number of three independent experiments were performed to ensure adequate statistical power. Student’s *t-*test was used for two-component comparisons. In the in vitro experiments, normal distribution was confirmed or assumed (for *n* < 5). Variance differences were analysed in comparisons between groups. For two-group analyses, Welch correction was applied for heteroscedastic models, and one-sample *t*-test analysis when one of the groups lacked variance (reference values). Two-tailed statistical analysis was applied for experimental design without predicted result, and one-tail for validation or hypothesis-driven experiments. The confidence level used for all the statistical analyses was of 0.95 (alpha value = 0.05).

## Results

**Fig. 1:**
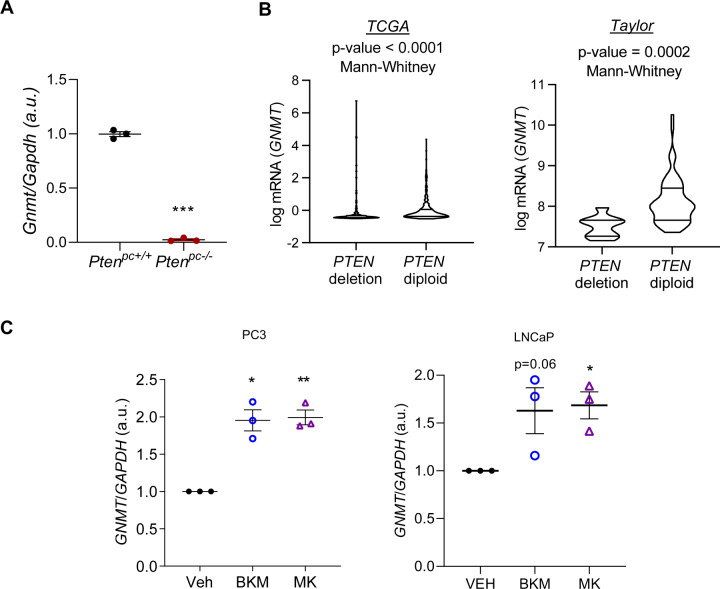
PTEN regulates *GNMT* expression. **A** *Gnmt* mRNA expression in *Pten*^*pc*−/−^ and *Pten*^*pc*+/+^ prostate tissue from 6-month-old mice (*n* = 3). **B** *GNMT* expression in PCa patient specimens with diploid or homozygous deletion of *PTEN* in two different datasets. Data was retrieved from www.cbioportal.org. **C** *GNMT* mRNA expression upon treatment (24 h) with Vehicle (Veh, DMSO), BKM120 (BKM, 5 µM) and MK2206 (MK, 500 nM) in PC3 and LnCaP cells (*n* = 3). Data are represented as mean with SEM (**A**, **C**) and violin plot (**B**). Statistic test: Student *T*-test (**A**); Mann–Whitney *U*-test (**B**) and One sample *T*-test (**C**). *p*, *p* value; *p < 0.05; **p < 0.01; ***p < 0.001.

To study the role of GNMT in PCa, we took advantage of a mouse model faithful to the human disease, the *Pten* conditional knockout mice, that we have previously characterized [7–9]. We analyzed prostate tissue from *Pten* prostate conditional knockout mice at the onset of invasive prostate carcinoma (PCa, 6 months old) [7]. To our surprise, cancerous lesions were accompanied by a profound reduction in *Gnmt* expression (Fig. 1A). We reasoned that this unexpected result could derive from the strong *Pten* loss-dependent PI3K stimulation, since this model has demonstrated high potential to uncover PI3K signaling-dependent molecular regulations [9]. PTEN is a negative regulator of PI3K signaling, which controls cancer cell biology at multiple levels [10]. To address this notion, we studied *GNMT* expression levels in human PCa specimens in which *PTEN* status was annotated [6, 38]. Importantly, *GNMT* mRNA levels were significantly lower in prostate tumors with *PTEN* deletion in two independent prostate cancer datasets [6, 38], thus supporting the results of our mouse model (Fig. 1B). To understand the signaling interplay and the mechanistic basis of PTEN-regulated *GNMT* expression, we employed genetic and pharmacological modulation in PC3 and LNCaP prostate cancer cell lines that present a complete loss of *PTEN*. In order to ascertain which components of this pathway are needed for the regulation of *GNMT*, we inhibited various proximal and distal effectors of PI3K, using BKM120 (BKM, Pan-PI3K inhibitor), MK2206 (MK, AKT inhibitor), and Rapamycin (R, mTOR complex 1 -mTORC1- inhibitor) (Supplementary Fig. S1A, B) [9]. Interestingly, drug treatment in two *PTEN*-deficient PCa cell lines revealed that *GNMT* expression was responsive to PI3K and AKT inhibition, but not to the blockade of mTORC1 (Fig. 1C, Supplementary Fig. S1C). Despite our efforts to detect GNMT protein levels, the antibodies tested exhibited limited sensitivity for endogenous GNMT in human prostate specimens, compared to ectopically-expressed protein (Data not shown).

AKT regulates the nuclear localization of the FOXO family of transcription factors [39]. Interestingly, the regulation of *GNMT* by FOXO has been reported in cells from different organisms, including the fruit fly and mouse models [40, 41]. We interrogated available chromatin immunoprecipitation (ChIP) data to further assess the binding of FOXO family factors to *GNMT* regulatory regions. The ENCODE consortium has generated a variety of resources using CRISPR-Cas9 technology to introduce ChIP-compatible tags prior to exon 1 of transcription factors and regulators [42]. Interestingly, ChIP-Seq data from tagged FOXO1 was available in ENCODE for the human HepG2 cell line. The results confirmed the presence of two putative binding sequences of FOXO1 in the *GNMT* promoter region (Supplementary Fig. 1D). To extend the implication of the chromatin-binding capacity of FOXO1 to *GNMT* promoter, we performed silencing experiments using FOXO1-targeting short hairpin RNA. As predicted, FOXO1 silencing significantly reduced the mRNA abundance of *GNMT* in human PC3 cells (Supplementary Fig. S1E). These results corroborate that AKT-FOXO1 axis controls, at least in part, the expression of *GNMT* in prostate cancer cells.

We next evaluated whether the regulation of *GNMT* by PI3K could be translated to non-stratified prostate cancer specimens. We interrogated a local cohort of benign prostate hyperplasia (BPH) and PCa human specimens [9] (clinical-pathological information available in Supplementary Table 1) and publicly available PCa datasets using Cancertool [43]. To our surprise, independent analysis of various cohorts revealed a profound lack of consistency, yielding no significant trend in *GNMT* expression in prostate tumors *vs*. non-cancerous tissue (Supplementary Fig. S2A–E), neither when comparing with normal tissue, when discriminating epithelial and stromal compartments [44] nor when accounting for tumor aggressiveness.

**Fig. 2:**
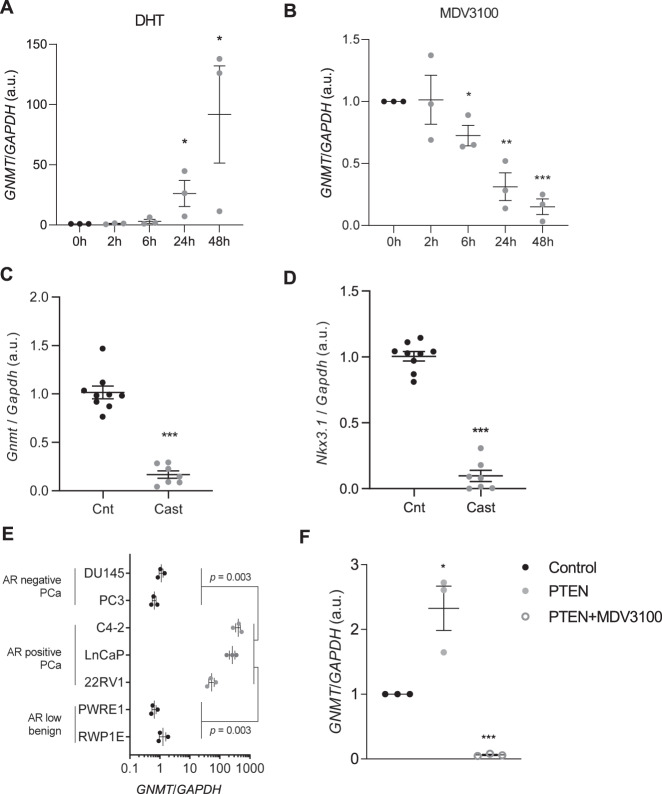
*GNMT* regulation by AR. **A**–**B** Effect of AR activation with 5α-dihydrotestosterone (DHT) (**A**) and AR inhibition by MDV3100 (**B**) on *GNMT* mRNA levels in a time course in LNCaP cells. **C**–**D** Effect of surgical castration of 6-month old *Pten*^*pc*−/−^ mice on *Gnmt* (**C**) and *Nkx3.1* (**D**) mRNA expression monitored by qPCR (*n* = 8–10) (Cnt Control non-castrated, Cast Castrated). **E** *GNMT* mRNA levels in prostate cancer cell lines with AR activity (represented with grey dots) and with low or no activity (black dots) (*n* = 3). **F** *GNMT* mRNA expression upon doxycycline-inducible expression (24 h) of YFP-PTEN in *PTEN*-deficient LNCaP prostate cancer cells in the presence or absence of the AR antagonist MDV3100 (*n* = 3). Values are represented as mean with SEM. Statistics: One sample *T*-test (**A**, **B**, **F**); Mann–Whitney *U*-test (**C**, **D**) and Student *T*-test with Welch correction (**E**). *p*, *p* value; *p < 0.05; **p < 0.01; ***p < 0.001.

The lack of consistency between our mechanistic findings and the clinical evidence pointed at the existence of other regulators of *GNMT* expression at play in PCa. We interrogated two main potential *GNMT* regulators, MYC and AR. On the one hand, we found that in vitro MYC inhibition using iBET [45] led to *GNMT* upregulation in two different cell lines (Supplementary Fig. S3A, B). These results are in agreement with studies reporting that this oncogene inhibits the expression of *GNMT* [20]. However, this level of regulation was not translated to an inverse correlation between *MYC* and *GNMT* in patient specimens (Supplementary Fig. S3C) [43]. On the other hand, we ascertained the influence of AR activation status on the expression of *GNMT*. We subjected AR-expressing (LNCaP) and non-expressing (PC3) cells to AR agonist and antagonist treatment. The evaluation of *GNMT* mRNA levels upon these perturbations confirmed that the expression of this enzyme is robustly activated by the nuclear receptor. LNCaP cells exhibited *GNMT* upregulation and repression upon treatment with Dihydrotestosterone (DHT) and MDV3100, respectively (Fig. 2A, B). In contrast, *GNMT* expression in PC3 cells remained unaffected upon exposure to these agents (Supplementary Fig. S4A). Furthermore, surgical castration in prostate-specific *Pten*-deficient mice with prostate cancer led to a profound decrease of *Gnmt* mRNA levels, consistent with the repression of a bona fide AR target, *Nkx3.1* (Fig. 2C, D). AR activity (by means of the expression of its target *KLK3*) was consistently correlated with the expression of *GNMT* in the majority of prostate cancer datasets analyzed (Supplementary Fig. S4B, C). However, we did not find a significant correlation between tumoral *GNMT* expression and circulating PSA levels, in line with the notion that blood PSA is associated to prostate mass rather than AR activity (Supplementary Fig. S4C).

The abundance of *GNMT* and *KLK3* mRNA was significantly higher in AR-expressing cell lines (22RV1, LnCaP, and C4-2), and both genes exhibited significant correlation (Fig. 2E, Supplementary Fig. S4D, E). We exploited these data further to confirm the results obtained through pharmacological and genetic means. We monitored the impact of *PTEN* status on *GNMT* expression in cell lines, to which end we focused on AR-negative cells to eliminate the dominant influence of androgen signaling. As predicted, *PTEN*-deficient PC3 cells exhibited lower *GNMT* mRNA abundance than *PTEN*-competent DU145 (GNMT expression in arbitrary units of 1.06 ± 0.27 in PC3 compared to 1.82 ± 0.49 in DU145; one-tailed Student *T*-test *p* = 0.039). In line with this notion, we observed that the re-expression of *PTEN* in LNCaP cells resulted in elevated *GNMT* expression, but this effect was abolished when AR was inhibited (Fig. 2F, Supplementary Fig. S5). Our results add complexity to the regulation of *GNMT* in PCa.

**Fig. 3:**
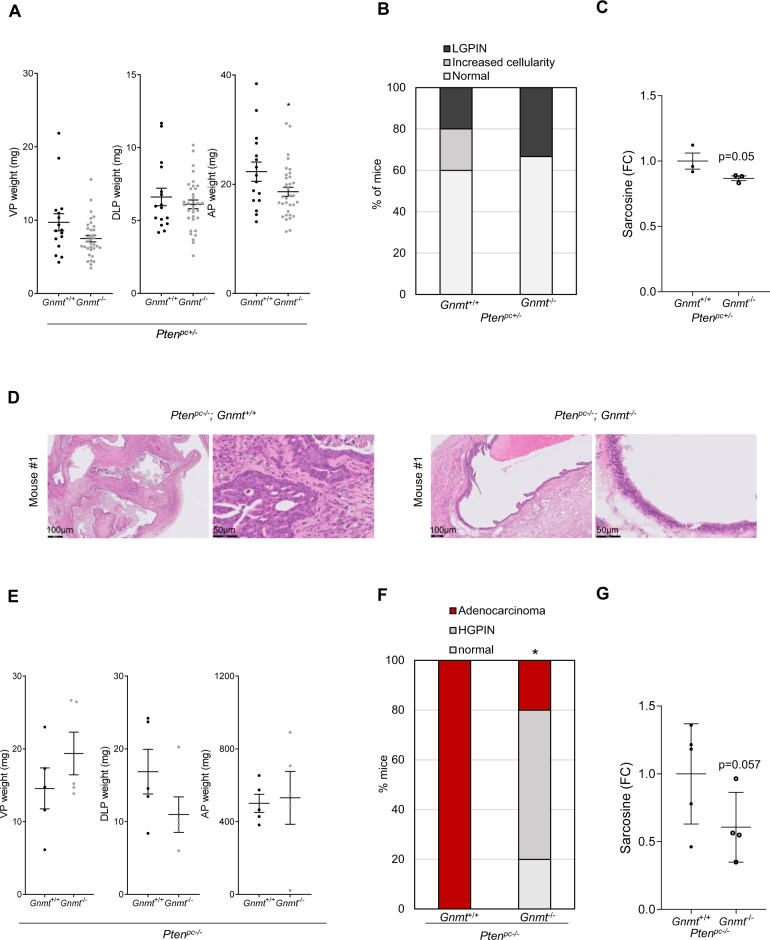
Gnmt is required for prostate cancer development. **A**–**C** Prostate lobe weights (VP, DLP, and AP) in *Pten*^*pc*+/−^;*Gnmt* ^+/+^ and *Pten*^*pc*+/−^;*Gnmt* ^−/−^ mice (2 individual lobes are presented per mouse, *n* = 8–16) (**A**); Histopathological characterization of the prostate tissue in *Pten*^*pc*+/−^;*Gnmt*^+/+^ and *Pten*^*pc*+/−^;*Gnmt* ^−/−^ mice (*n* = 8–16) (**B**); Sarcosine abundance in anterior prostate (AP) lobes in the indicated genotypes measured by LC/MS (*n* = 3) (**C**). **D**–**G** Representative histological images of Hematoxylin Eosin (H&E) staining (100X and 400X) in *Pten*^*pc*−/−^;*Gnmt*^+/+^ and *Pten*^*pc−*/−^;*Gnmt*^−/−^ mouse prostates (**D**); Comparison of different prostate lobe weights (VP, DLP, and AP) (represented in the graph with 2 different individual lobes) (*n* = 4–5) *Pten*^*pc*−/−^;*Gnmt*^+/+^ and *Pten*^*pc*−/−^;*Gnmt*^−/−^ mice (**E**); Histopathological characterization of the prostate tissue (*n* = 5) (**F**) and Sarcosine amount in anterior prostate (AP) lobes measured by LC/MS (*n* = 4–5) (**G**) in *Pten*^*pc*−/−^;*Gnmt*^+/+^ and *Pten*^*pc*−/−^;*Gnmt*^−/−^ mice (*n* = 5). VP Ventral prostate, DLP Dorsolateral prostate, AP Anterior prostate, LGPIN Low-grade Prostatic Intraepithelial Neoplasia, HGPIN High grade Prostatic Intraepithelial Neoplasia. Statistics: One tail Mann-Whitney *U* test (**A** left and middle panels, **C**, **E**, **G**), Two tailed Student *T* test (right panel), and Chi square (**F**) was used for data analysis. *p*, *p* value; *p < 0.05.

Independent studies suggest that GNMT either promotes or suppresses PCa aggressiveness [14, 15, 17, 19]. The opposing regulation of this metabolic enzyme by androgens, MYC, and PI3K made difficult to anticipate its role in PCa. The activation of *GNMT* expression by androgens would be consistent with a tumor-promoting role, whereas the inhibition by the MYC and PI3K pathways would argue in the opposite direction. We chose to experimentally discern its contribution to various aspects of PCa biology. To this end, we took advantage of a germline *Gnmt*-deficient mouse model [13] and evaluated the impact of deleting this enzyme on prostate histology. The evaluation of *Gnmt* knockout mice for up to 1-year did not reveal neither changes in prostate mass nor pathological alterations compatible with cancer (Supplementary Fig. S6A–C). In line with this notion, we did not detect any significant metabolic alterations in the prostate tissue of these mice, neither at 8 months of age (Supplementary Fig. S6D and Supplementary Table 2) nor at the experimental end point (12 months of age, Supplementary Table 3). Similarly, *Gnmt* deletion did not impact on the prostate mass or pathology of mice in a prostate conditional *Pten* heterozygous background. *Pten* heterozygous mice exhibited PIN lesions at low incidence, as reported (Fig. 3A, B, Supplementary Fig. S7A) [25], and this phenotype remained unmodified when *Gnmt* was deleted. Interestingly, we found a reduction in sarcosine abundance in *Gnmt*-deleted tissues, and a significant increase in betaine (Fig. 3C; Supplementary Fig. S7B; Supplementary Table 4 for additional metabolites measured). In line with our previous work and other studies, conditional homozygous *Pten* deletion in the prostate epithelium resulted in the development of PCa [25]. This phenotype was fully penetrant in this mixed genetic background and at 12 months of age. *Gnmt* deletion in this model resulted in a distinct histological phenotype, with a remarkable decrease in the incidence of adenocarcinoma (Fig. 3D–F; Supplementary Fig. S8A). *Pten*-*Gnmt* double knockout mice retained PIN lesions that did not progress to cancer, whereas the overall tissue weight remained unaffected. At the metabolic level, *Gnmt* deletion resulted in a modest reduction in prostatic sarcosine levels (Fig. 3G) together with a significant perturbation of putrescine and methionine (Supplementary Fig. S8B; Supplementary Table 5 for additional metabolites measured).

**Fig. 4:**
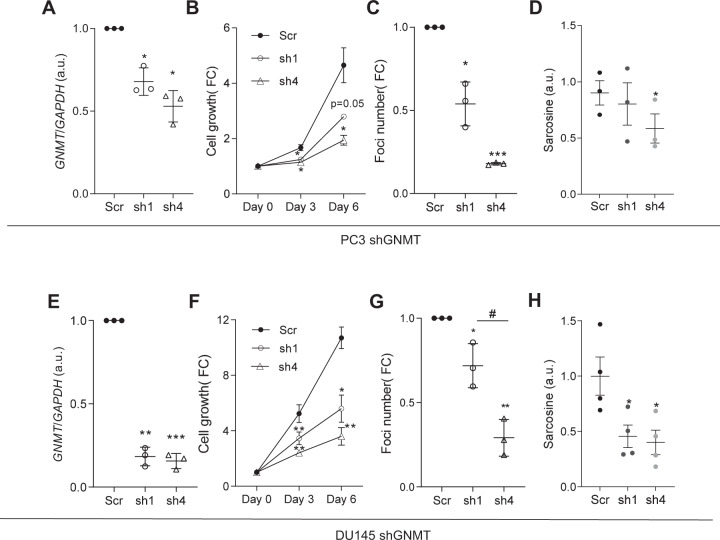
Impact of genetic *Gnmt* modulation in prostate cancer pathogenesis. **A**–**D**, Effect of *GNMT* silencing on mRNA levels (**A**), cell number (**B**), foci formation (**C**), and sarcosine levels by LC/MS (**D**) in PC3 cells (*n* = 3). **E**–**H**, Effect of *GNMT* silencing on mRNA levels (**E**), cell number (**F**), foci formation (**G**), and sarcosine levels by LC/MS (**H**) in DU145 cells (*n* = 3–4). Data are represented as mean with SEM. scr: scramble short hairpin; sh1 and sh4: two different short-hairpins targeting *GNMT*; *p*, *p* value; *p < 0.05; **p < 0.01; ***p < 0.001. Statistic test: One Sample (**A**, **C**, **E**, **G**) and or one-tailed paired (**B**, **D**, **F**, **H**) Student *T*-test was used for data analysis.

The results in vivo are derived from a mouse model in which *Gnmt* was deleted systemically. In order to address whether the effect of *Gnmt* deletion was based on prostate cancer cell-autonomous effects, we silenced *GNMT* in three independent PCa cell lines with differing AR status and *GNMT* levels (PC3, DU145, and LNCaP) using two different short hairpin RNAs (Fig. 4A–H; Supplementary Fig. S9A–D). *GNMT* silencing had a significant impact on cell number, with a reduction in two-dimensional growth and foci formation (Fig. 4B, C, F, G; Supplementary Fig. S9B). In addition, we could confirm that the reduction in *GNMT* levels resulted in an overall decrease in its metabolic product, sarcosine, in PC3 and DU145 cells (Fig. 4D, H), in the absence of consistent alterations in a large panel of metabolites (Supplementary Fig. S9C). Of note, *GNMT* silencing elicited an increase in the apoptotic marker cleaved PARP in LnCaP and PC3 cells, in line with previous reports [46] (Supplementary Fig S9D). Altogether, the results in cell lines corroborate the in vivo results and demonstrate the requirement of GNMT for the proper maintenance of tumorigenic features.

## Discussion

Experimental systems are instrumental to provide a good understanding of biological processes relevant to human health. However, different approaches can yield apparently conflicting results. In the context of GNMT, there is sufficient evidence to support its regulation by androgen signaling [21, 22]. In addition, in vitro and xenograft approaches provided support for a positive role of GNMT in the regulation of specific PCa features [14, 15], despite the fact that some studies raised controversy around its potential tumor-suppressive nature [19]. Similarly, the analysis of human specimens has resulted in discordant observations, from upregulation in PCa [14], to downregulation in cancerous specimens [19]. Another line of evidence suggests that polymorphisms in *GNMT* gene are associated to higher risk of suffering PCa [19, 23], although the functional consequences of those genetic alterations remain obscure. Our work provides a comprehensive analysis of the role and regulation of *GNMT* in PCa. We integrate and rationalize the mechanism behind the discordant regulation of this enzyme in human and murine specimens of PCa. In addition, we provide solid evidence of the requirement of GNMT for the development of PCa using genetic mouse models complemented with genetic manipulation in cellular systems.

**Fig. 5:**
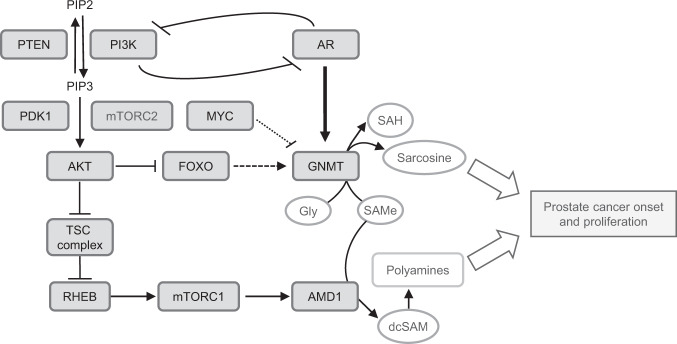
Integrative view of GNMT regulation in prostate cancer. Schematic representation of Methionine cycle, Polyamine metabolism, and PI3K-mTOR axis interaction. PIP2 phosphatidylinositol 4,5-bisphosphate, PIP3 phosphatidylinositol 3,4,5-trisphosphate, PDK1 Phosphoinositide-dependent kinase 1, TSC tuberous sclerosis protein complex, RHEB Ras homolog enriched in brain, Gly Glycine.

The regulation of *GNMT* in PCa is dominated by the action of AR. This gene contains androgen response elements (AREs) that makes it responsive to the activity of the nuclear receptor [15, 21, 22]. Yet, this enzyme is not consistently upregulated in PCa according to our comprehensive analysis, which adds complexity to the already contradictory published reports [14, 19]. Our results depicting the strong correlation between *GNMT* and *KLK3*, together with the characteristic pattern of expression of this enzyme in prostate cell lines supports the notion that androgens are the main signal controlling *GNMT* expression, but this level of regulation co-exists with the action of other oncogenic signaling pathways (Fig. 5).

Our study demonstrates that other PCa-relevant signals influence *GNMT* expression. On the one hand, we observed an upregulation of this enzyme upon pharmacological blockade of MYC signaling with iBET. On the other hand, we documented the negative regulation of *GNMT* mRNA levels by the PI3K pathway. PI3K activation, prominently due to alterations in *PTEN*, is a relevant oncogenic signal in cancer, and specifically in prostate tumors [1, 8, 14] (Fig. 5). The fact that our mouse model exhibited an unexpected reduction in *Gnmt* expression prompted us to study the relationship between this event and the genetic nature of the tumors, driven by prostate-specific activation of the PI3K pathway. Our analysis in the mouse model, complemented with pharmacological analyses and associative studies in PCa specimens demonstrates that this metabolic enzyme is under negative regulation of the PI3K axis. We ruled out that this event would be mTORC1-dependent, in contrast to our observations with AMD1, another enzyme associated to SAMe metabolism [9]. Interestingly, PI3K and AKT inhibitors incremented *GNMT* expression, suggesting that the regulation stems from an AKT effector. There are two possible explanations for this observation. First, the PI3K and AR exhibit a mutually inhibitory cross talk [47, 48]. It is therefore plausible that inhibition of PI3K-AKT would further enhance AR activity and promote *GNMT* expression, as reported for MYC [20]. However, the fact that we could replicate the increase in *GNMT* with PI3K inhibitors in AR-negative cells (PC3) suggests that another mechanism exists beyond the regulation by AR. Second, recent studies in different experimental systems suggest that FOXO (a transcription factor under negative regulation by AKT) regulates the expression of *GNMT* [40, 41]. These studies are consistent with data generated in the context of ENCODE consortium, where FOXO1 is shown to bind to *GNMT* promoter region in HepG2 cells [49]. In this line, we confirmed the transcriptional control of *GNMT* by FOXO1 using shRNA against the transcription factor in prostate cancer cells. This mechanism is consistent with our results in vitro and in vivo. Altogether, our data reveal that *GNMT* expression is determined by the equilibrium between androgen and PI3K-dependent signals.

Our results provide further demonstration for the requirement of GNMT in the acquisition of cancerous features by prostate cells. To date, the association of GNMT to PCa biology is based on the use of cell lines and correlative human analyses [14, 15, 19]. We provide unprecedented evidence showing that expression of GNMT is required for the onset of invasive prostate carcinoma. Ablation of *Gnmt* in mice with conditional deletion of *Pten* in the prostate epithelium prevented the development of full-blown prostate carcinoma. Our results suggest that despite the PI3K-dependent downregulation of *Gnmt* in the murine prostate, basal expression of this gene is essential for PCa to emerge.

GNMT catalyzes the production of sarcosine and S-adenosylhomocysteine (SAH) from glycine and SAMe [12]. It has been proposed that sarcosine production accounts for the majority of pro-tumorigenic activities of GNMT [17]. In our study, sarcosine is the metabolite that best illustrates the changes in *GNMT* expression, which supports the notion that this metabolite could be responsible for its effects [15, 16, 18]. Sarcosine production is a suggested non-invasive PCa biomarker [15]. However, these results remain controversial, according to various follow-up studies in different PCa cohorts [50]. Moreover, many groups have attempted the development of methods to monitor sarcosine abundance [51–53], and combination of sarcosine with other metabolites has been tested in order to test its prognostic potential [54]. The opposed regulation of *GNMT* expression by AR *vs*. MYC and PI3K would suggest that the levels of sarcosine in PCa patients could be influenced by *PTEN* and *MYC* status. Therefore, it remains to be evaluated whether the potential of sarcosine as a PCa biomarker would benefit from *PTEN* and/or *MYC* status-based patient stratification.

PI3K activation elicits the concomitant repression of *GNMT* and activation of AMD1 [9]. This dual regulation suggests that the oncogenic signaling pathway could control the fate of SAMe by downregulating its demethylation in favor of its decarboxylation. Interestingly, GNMT perturbation alone does not affect polyamine biosynthesis based on our analysis of murine specimens and cell lines. These data is in line with the lack of oncogenic prostate phenotype observed in the mouse model upon *Gnmt* deletion alone, which would be expected if polyamine production would increase.

This study provides evidence of the requirement of GNMT for PCa pathogenesis, and reveals an unprecedented PI3K-dependent regulation that broadens our perspective around the prostate-intrinsic signals that govern SAMe metabolism.

## Supplementary information

Supplementary figures and legends

Suppl Table 1

Suppl Table 2

Suppl Table 3

Suppl Table 4

Suppl Table 5
