# Supplementary material for: PI3K-regulated Glycine N-methyltransferase is required for the development of prostate cancer
Source: Oncogenesis. 2022 Feb 23;11(1):10. doi: 10.1038/s41389-022-00382-x (PMC8866399; doi:10.1038/s41389-022-00382-x)
Supplement: Supplementary file 1 — Supplementary figures and legends [file 41389_2022_382_MOESM1_ESM.pdf]

SUPPLEMENTARY FIGURE LEGENDS

Supplementary Figure 1.

a-b, Effect of PI3K-mTORC1 axis inhibition on AKT and RpS6 phosphorylation in PC3 at 20h (a) and LnCaP at the indicated time points (b) upon treatment with Vehicle (Veh, DMSO), BKM120 (5µM), MK2206 (MK, 500nM) and Rapamycin (R, 20nM).

c, *GNMT* mRNA expression upon treatment (24h) with Vehicle (Veh, DMSO) and Rapamycin (R, 20nM) in PC3 and LnCaP cells (n=3).

d, Genomic overview of the promoter region of human *GNMT* gene. The figure shows regulatory elements located within the promoter region of the GNMT gene (chr6:42959219-42962219, hg38) including FOXO1 binding sites (based on ChIP-Seq experiments), as well as promoter and enhancer-like regulatory elements (based on DNase and histone modification ChIP-Seq experiments) from ENCODE.

e, GNMT and FOXO1 mRNA levels upon FOXO1 silencing (n=3).

Statistic test: One sample T-test (d).

p, p-value; *, $p<0.05$; **, $p<0.01$.

Supplementary Figure 2.

a, *GNMT* mRNA expression in human prostate tissue specimens of BPH (n=6) and prostate cancer (n=16).

b, *GNMT* mRNA expression levels in three different PCa datasets (N:normal; PCa: Prostate Cancer).

c, *GNMT* mRNA expression levels in three different PCa datasets (N:normal; PT: Primary Tumor; M: Metastasis).

d, *GNMT* mRNA expression levels in three different PCa datasets (GS: Gleason Score).

e, *GNMT* mRNA expression levels in PCa vs normal adjacent tissue in epithelium (left) and stroma (right) (Tyekucheva, *et* al., 2017; Nature communications).

Statistic test: Student T-test (a, b, e) and ANOVA (c, d).

p, p-value; *, $p<0.05$.

Supplementary Figure 3.

a,b, *GNMT* mRNA expression (left) upon iBET treatment in DU145 (a) and PC3 (b) cells. *FASN* (right) gene expression is used as a readout of target inhibition.

c, Correlation of *GNMT* and *MYC* mRNA expression in four different prostate cancer datasets.

Values are expressed as mean with SEM.

Statistics: One sample T-test (a,b).

Supplementary Figure 4.

a, Effect of AR activation with 5α-dihydrotestosterone (DHT) (a, left) and AR inhibition by MDV3100 (b, right) on *GNMT* mRNA levels in PC3 cells.

b, Correlation analysis of *GNMT* and *KLK3* mRNA expression in four prostate cancer datasets in primary tumors.

c, Correlation analysis of *GNMT* and *KLK3* mRNA expression in prostate cancer tissue specimens (left panels) and blood PSA levels (right panels) from Basurto cohort.

d, *KLK3* mRNA levels in prostate benign and cancer cell lines (n=3).

e, Correlation analysis of *GNMT* and *KLK3* mRNA expression in a panel of prostate cancer cell lines from (c).

Values are represented as mean with SEM (a, c, d, e). Statistics: One sample T-test (a), Spearman correlation (b, c, e) and Student T test with Welch correction (d). p, p-value; *, $p<0.05$; **, $p<0.01$.

Supplementary Figure 5.
Representative western blot monitoring cleaved PARP and AKT phosphorylation upon doxycycline-inducible expression (24 h) of YFP–PTEN in *PTEN*-deficient LNCaP prostate cancer cells (*n* = 3 independent experiments).

Supplementary Figure 6.
a-d, Representative histological images of Hematoxylin Eosin (H&E) staining (100X and 400X) in *Gnmt* +/+ and *Gnmt* -/- mouse prostates (a); Comparison of different prostate lobe weights (VP, DLP and AP) (each one represented in the graph by 2 different individual lobes) from *Gnmt* +/+ and *Gnmt* -/- mice (n=16) (b); Histopathological characterization of prostate tissue from *Gnmt* +/+ and *Gnmt* -/- mice (n=16) (c); LC/MS analysis of methionine cycle and polyamine pathway metabolites in prostates from *Gnmt*$^{-/-}$ compared to *Gnmt*$^{+/+}$ mice (n=4) (d). Dash line indicates relative metabolite abundance in *Gnmt*$^{+/+}$ (WT) mice.
Data are represented as mean with SEM. Panel B: VP Student T Test with Welch´s correction; DLP Student T Test, AP Mann Whitney. Panel D, Mann Whitney.
SAM: S-Adenosylmethionine; SAH: S-Adenosylhomocysteine; MTA: 5´ Methylthioadenosine.

Supplementary Figure 7.
a, Representative histological images of Hematoxylin Eosin (H&E) staining (100X) in *Pten*$^{pc+/-}$ *Gnmt*$^{-/-}$ and *Pten*$^{pc+/-}$ *Gnmt*$^{+/+}$ mice.
b, LC/MS analysis of methionine cycle and polyamine pathway metabolites in prostates from *Pten*$^{pc+/-}$ *Gnmt*$^{-/-}$ relative to *Pten*$^{pc+/-}$ *Gnmt*$^{+/+}$ (left) (n=3) mice. Dash line indicates relative metabolite abundance in *Pten*$^{pc+/-}$ *Gnmt*$^{+/+}$ mice.
Data are represented as mean with SEM. Statistic tests: Mann Whitney test.
SAM: S-Adenosylmethionine; SAH: S-Adenosylhomocysteine; MTA: 5´ Methylthioadenosine.

Supplementary Figure 8.
a, Representative histological images of Hematoxylin Eosin (H&E) staining (100X and 400X) in *Pten*$^{pc-/-}$ *Gnmt*$^{-/-}$ and *Pten*$^{pc-/-}$ *Gnmt*$^{+/+}$ mice.
b, LC/MS analysis of methionine cycle and polyamine pathway metabolites in prostates from *Pten*$^{pc-/-}$ *Gnmt*$^{-/-}$ and *Pten*$^{pc-/-}$ *Gnmt*$^{+/+}$ (n=4-5) mice. Dash line indicates relative metabolite abundance in *Pten*$^{pc-/-}$ *Gnmt*$^{+/+}$ mice.
Data are represented as mean with SEM. MannWhitney test was used for data analysis.
SAM: S-Adenosylmethionine; SAH: S-Adenosylhomocysteine; MTA: 5´ Methylthioadenosine.

Supplementary Figure 9.
a-b, Effect of *GNMT* genetic silencing (a) on cell number (b) in LnCaP cells (n=3).
c, LC/MS analysis of methionine cycle and polyamine pathway metabolites in *GNMT* silenced PC3 (left) and DU145 (right) cells. Dash line indicates relative metabolite abundance in scramble shRNA cells.
d, Analysis of the apoptotic marker cleaved PARP in *GNMT* silenced PC3 and LnCaP cells.
Data are represented as mean with SEM (a, b, c).
Statistics: One sample T-test (a), paired Student T test (b) and One Sample T test (c).
p, p-value; *, $p<0.05$; **, $p<0.01$; ***, $p<0.001$
SAM: S-Adenosylmethionine; SAH: S-Adenosylhomocysteine; MTA: 5´ Methylthioadenosine; Putr: Putrescine; Spd: Spermidine; Spm: Spermine; Ac-Spm: Acetylated Spermine; -D: -doxycicline; +D: +doxycicline.

Supplementary Table 1. Clinical-pathological characteristics of patients analyzed in the Basurto cohort. BPH, benign prostate hyperplasia.

Supplementary Table 2. Metabolic alterations in *Gnmt*$^{-/-}$ *vs. Gnmt* $^{+/+}$ mice at 8 months of age

Supplementary Table 3. Metabolic alterations in *Gnmt*$^{-/-}$ *vs. Gnmt* $^{+/+}$ mice at 12 months of age

Supplementary Table 4. Metabolic alterations in *Pten*$^{pc+/-}$ *Gnmt*$^{-/-}$ vs. *Pten*$^{pc+/-}$ *Gnmt*$^{+/+}$ mice

Supplementary Table 5. Metabolic alterations in *Pten*$^{pc-/-}$ *Gnmt*$^{-/-}$ and *Pten*$^{pc-/-}$ *Gnmt*$^{+/+}$ mice

Figure S1

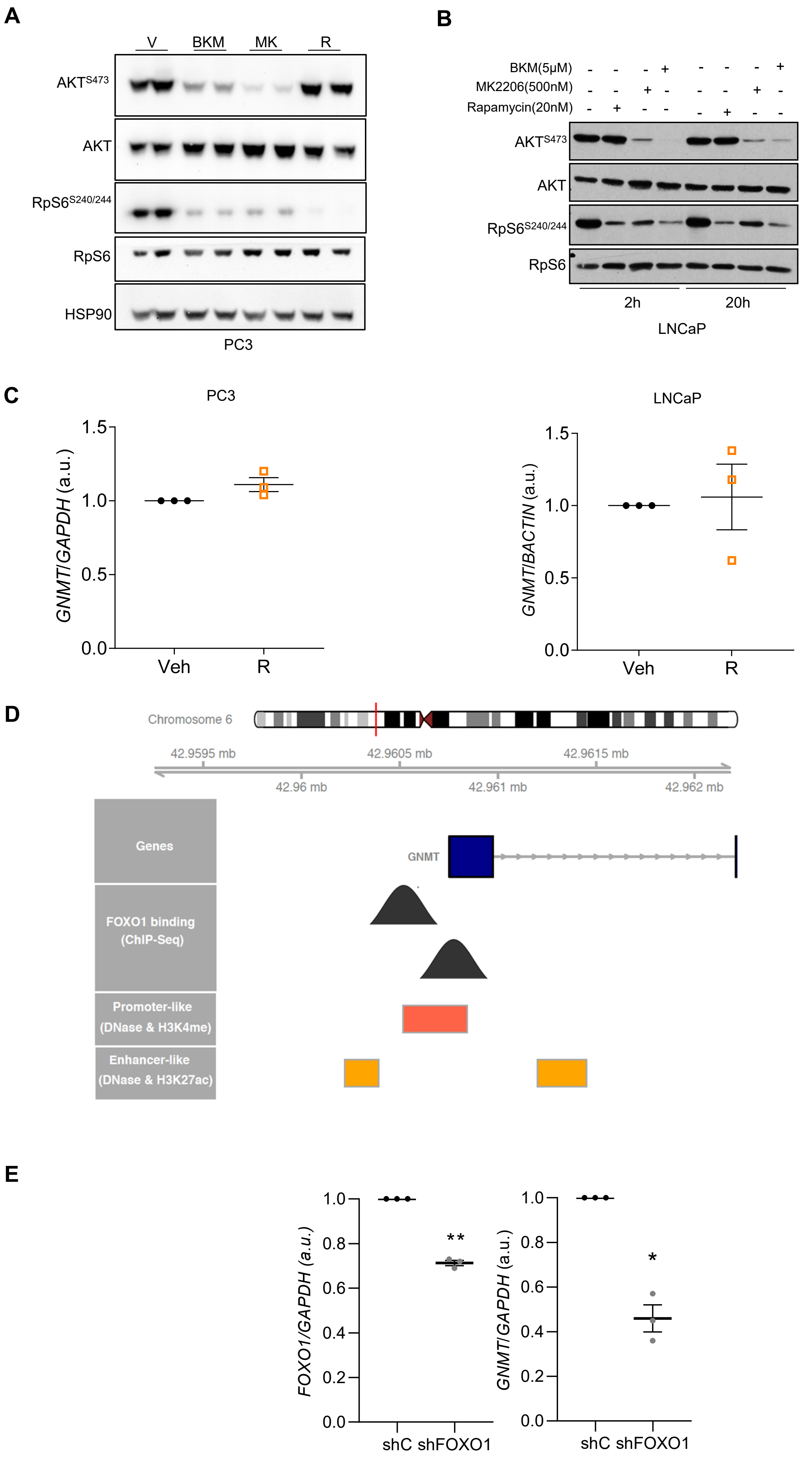
A
V
BKM
MK
R
AKT^S473
AKT
RpS6^S240/244
RpS6
HSP90
PC3
B
BKM(5μM)
MK2206(500nM)
Rapamycin(20nM)
AKT^S473
AKT
RpS6^S240/244
RpS6
2h
20h
LNCaP
C
PC3
LNCaP
GNMT/GAPDH (a.u.)
GNMT/BACTIN (a.u.)
1.5
1.0
0.5
0.0
Veh
R
D
Chromosome 6
42.9595 mb
42.9605 mb
42.9615 mb
42.96 mb
42.961 mb
42.962 mb
Genes
GNMT
FOXO1 binding (ChIP-Seq)
Promoter-like (DNase & H3K4me)
Enhancer-like (DNase & H3K27ac)
E
FOXO1/GAPDH (a.u.)
GNMT/GAPDH (a.u.)
1.0
0.8
0.6
0.4
0.2
0.0
**
*
shC shFOXO1

Figure S2

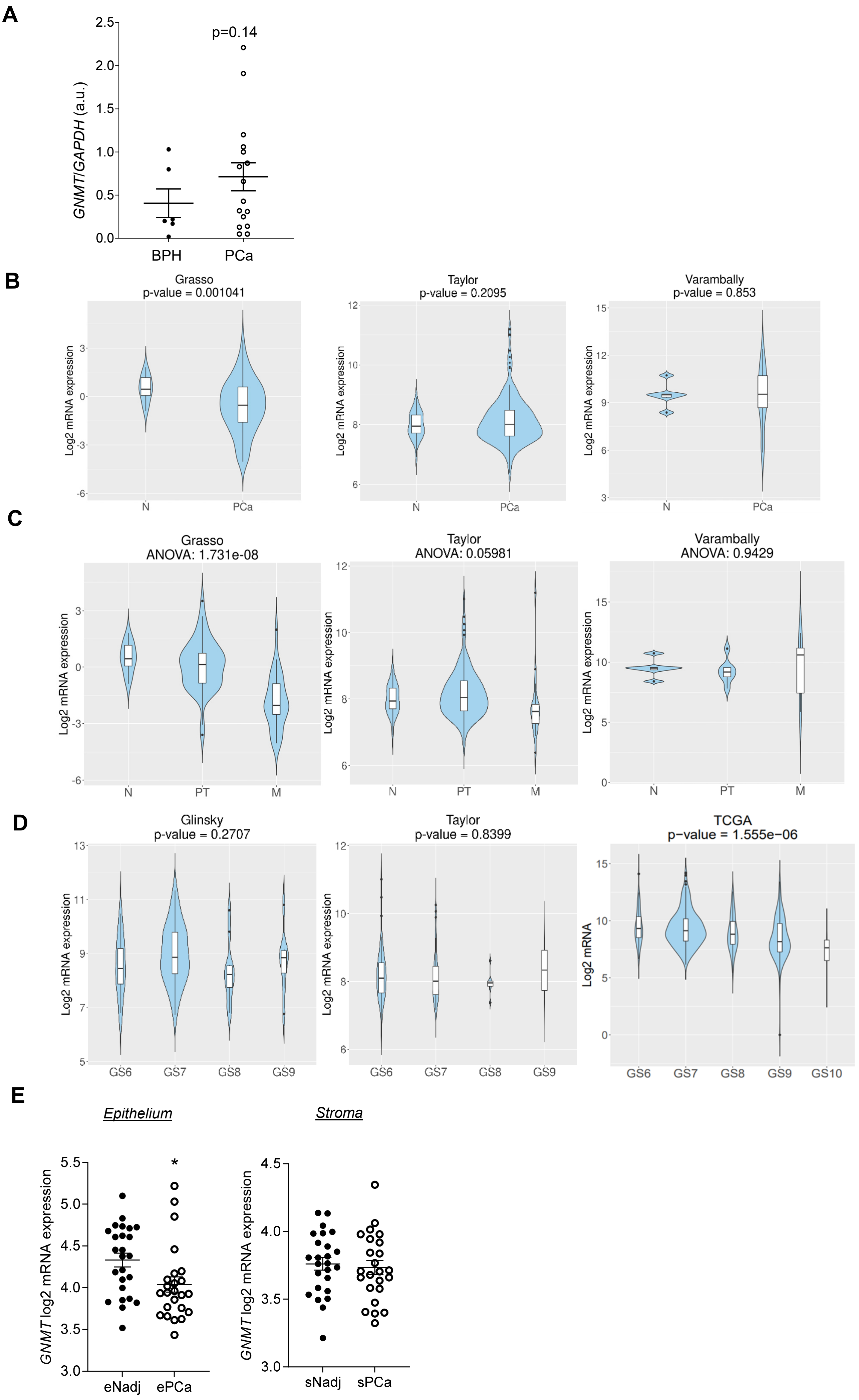
A
p=0.14
GNMT/GAPDH (a.u.)
2.5
2.0
1.5
1.0
0.5
0.0
BPH
PCa
B
Grasso
p-value = 0.001041
Taylor
p-value = 0.2095
Varambally
p-value = 0.853
Log2 mRNA expression
N
PCa
C
Grasso
ANOVA: 1.731e-08
Taylor
ANOVA: 0.05981
Varambally
ANOVA: 0.9429
Log2 mRNA expression
N
PT
M
D
Glinsky
p-value = 0.2707
Taylor
p-value = 0.8399
TCGA
p−value = 1.555e−06
Log2 mRNA expression
Log2 mRNA
GS6
GS7
GS8
GS9
GS10
E
Epithelium
Stroma
*
GNMT log2 mRNA expression
eNadj
ePCa
sNadj
sPCa

Figure S3

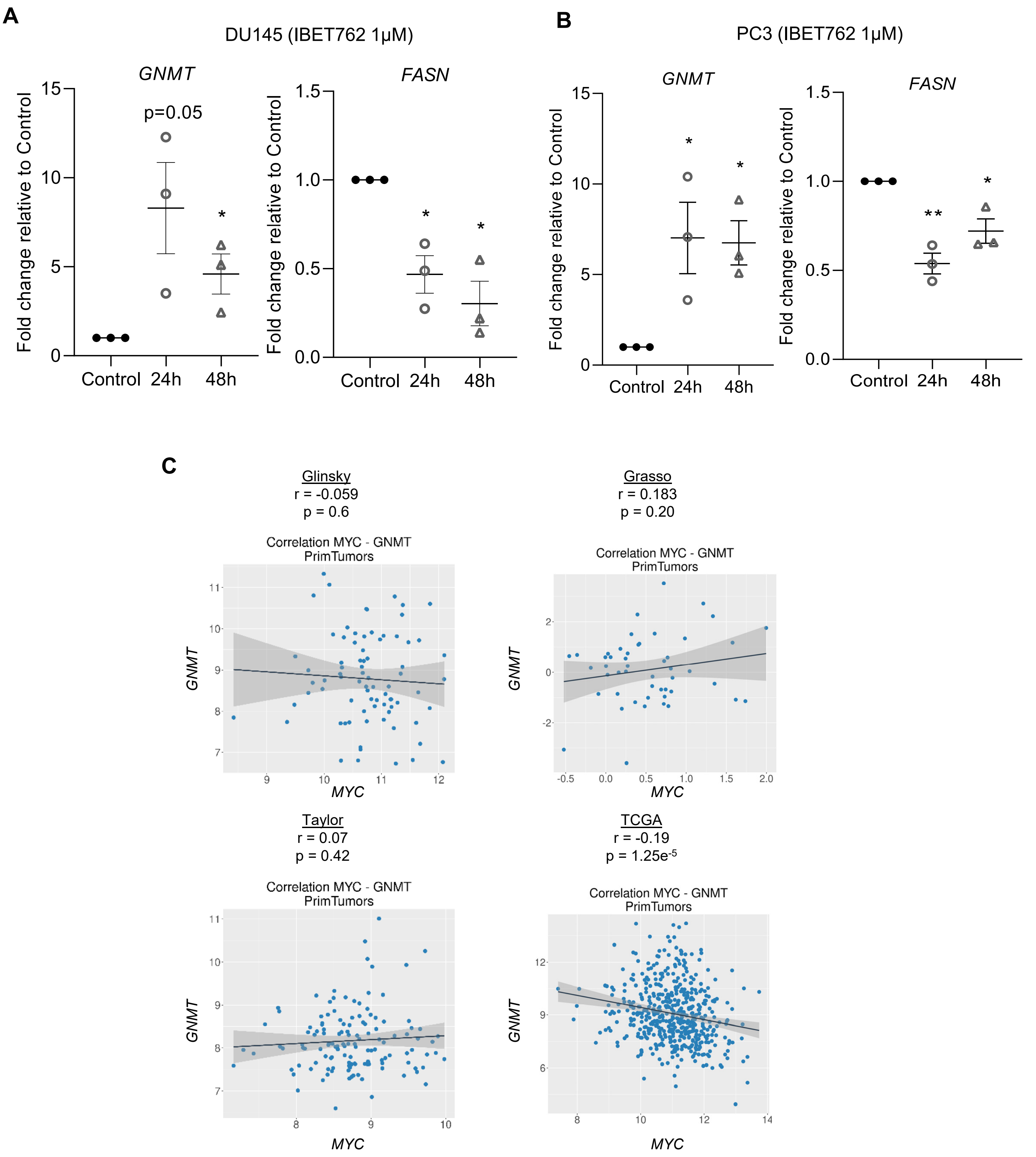
A
DU145 (IBET762 1μM)
GNMT
p=0.05
FASN
Fold change relative to Control
Control
24h
48h
B
PC3 (IBET762 1μM)
GNMT
FASN
C
Glinsky
r = -0.059
p = 0.6
Grasso
r = 0.183
p = 0.20
Taylor
r = 0.07
p = 0.42
TCGA
r = -0.19
p = 1.25e-5
Correlation MYC - GNMT
PrimTumors
GNMT
MYC

Figure S4

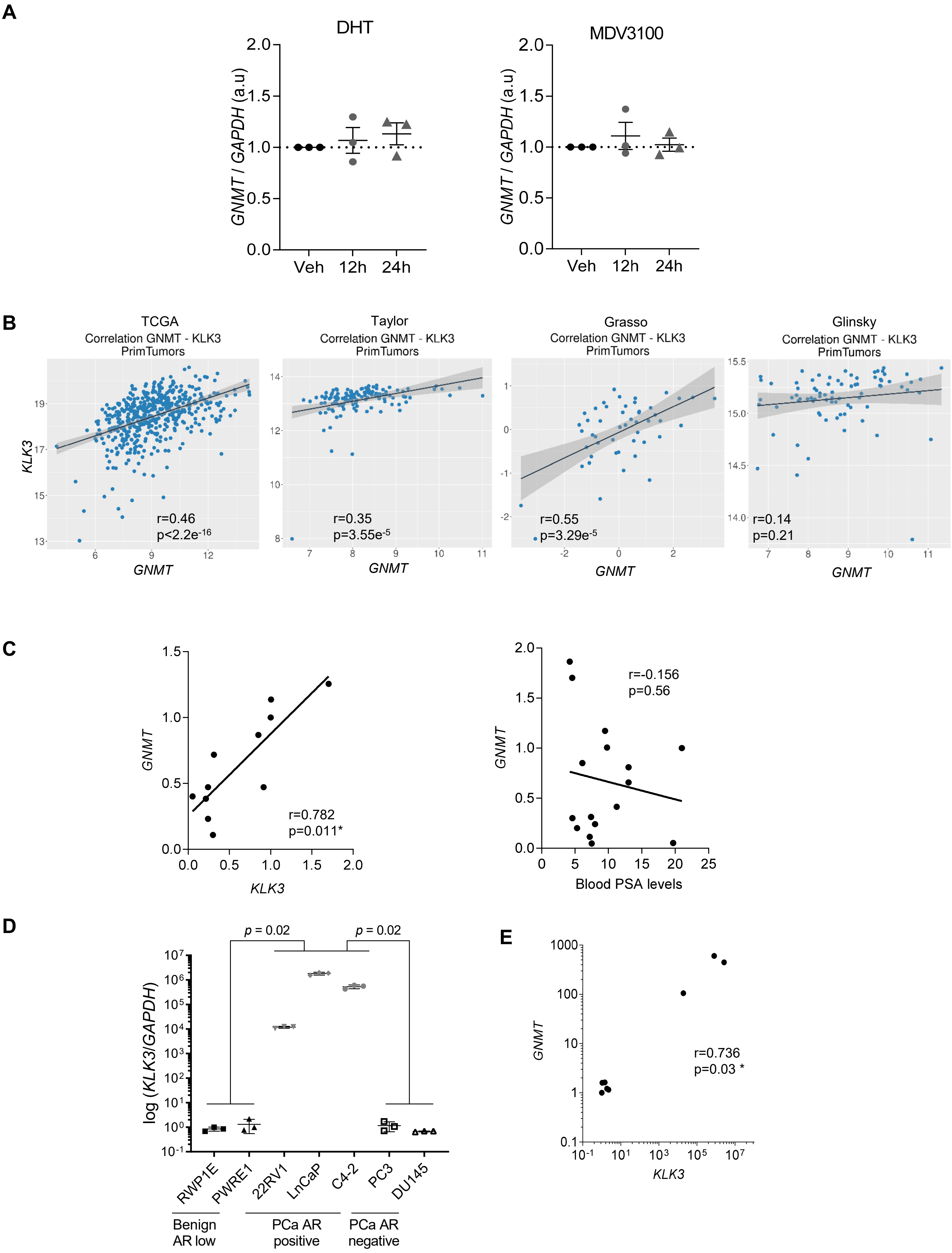
A
DHT
MDV3100
GNMT / GAPDH (a.u)
Veh
12h
24h
B
TCGA
Taylor
Grasso
Glinsky
Correlation GNMT - KLK3
PrimTumors
KLK3
GNMT
r=0.46
p<2.2e-16
r=0.35
p=3.55e-5
r=0.55
p=3.29e-5
r=0.14
p=0.21
C
GNMT
KLK3
r=0.782
p=0.011*
r=-0.156
p=0.56
Blood PSA levels
D
p = 0.02
p = 0.02
log (KLK3/GAPDH)
RWP1E
PWRE1
22RV1
LnCaP
C4-2
PC3
DU145
Benign
AR low
PCa AR
positive
PCa AR
negative
E
GNMT
KLK3
r=0.736
p=0.03 *

Figure S5

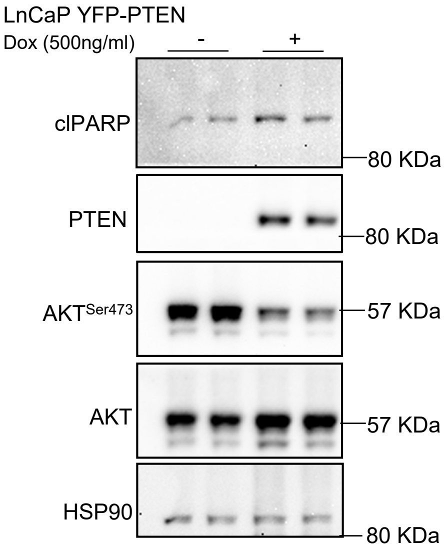

LnCaP YFP-PTEN
Dox (500ng/ml)
-
+
clPARP
80 KDa
PTEN
80 KDa
AKT^Ser473
57 KDa
AKT
57 KDa
HSP90
80 KDa

Figure S6

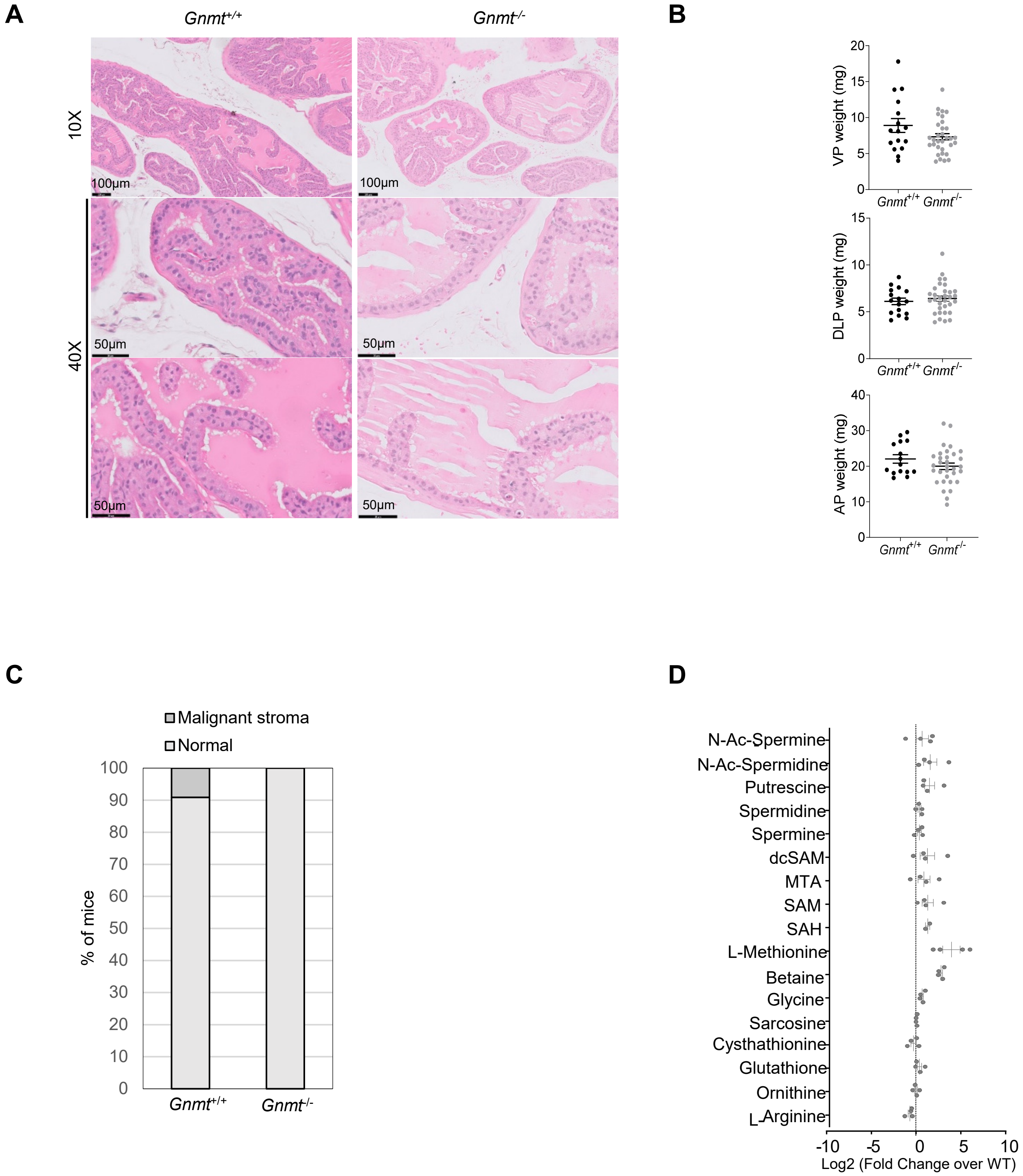
A
Gnmt+/+
Gnmt-/-
10X
40X
100µm
100µm
50µm
50µm
50µm
50µm
B
VP weight (mg)
DLP weight (mg)
AP weight (mg)
Gnmt+/+ Gnmt-/-
C
Malignant stroma
Normal
% of mice
Gnmt+/+
Gnmt-/-
D
N-Ac-Spermine
N-Ac-Spermidine
Putrescine
Spermidine
Spermine
dcSAM
MTA
SAM
SAH
L-Methionine
Betaine
Glycine
Sarcosine
Cysthathionine
Glutathione
Ornithine
L-Arginine
Log2 (Fold Change over WT)

Figure S7

A

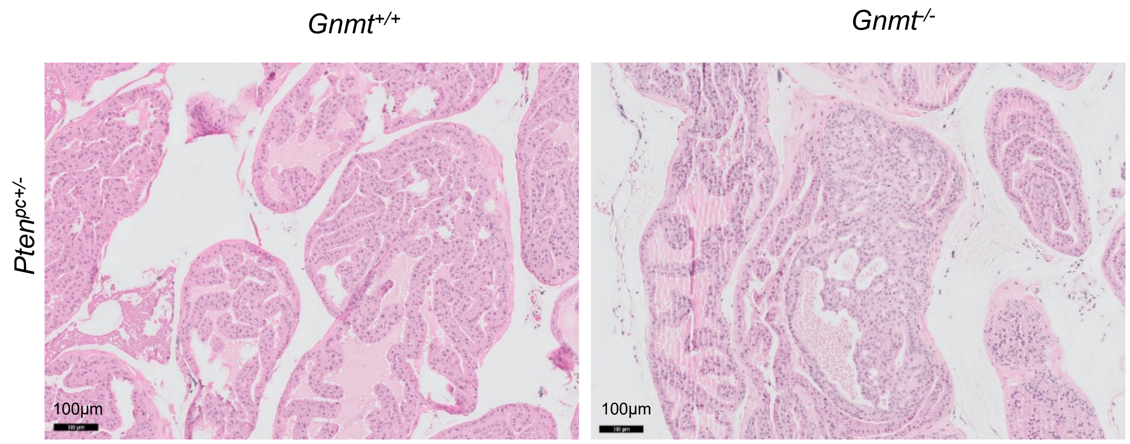

Gnmt+/+
Gnmt-/-
Ptenpc+/-
100µm
100µm

B

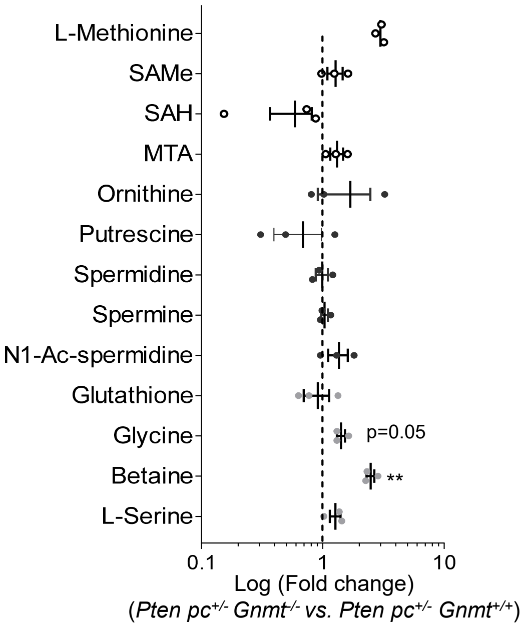

L-Methionine
SAMe
SAH
MTA
Ornithine
Putrescine
Spermidine
Spermine
N1-Ac-spermidine
Glutathione
Glycine
Betaine
L-Serine
p=0.05
**
0.1
1
10
Log (Fold change)
(Pten pc+/- Gnmt-/- vs. Pten pc+/- Gnmt+/+)

Figure S8

A

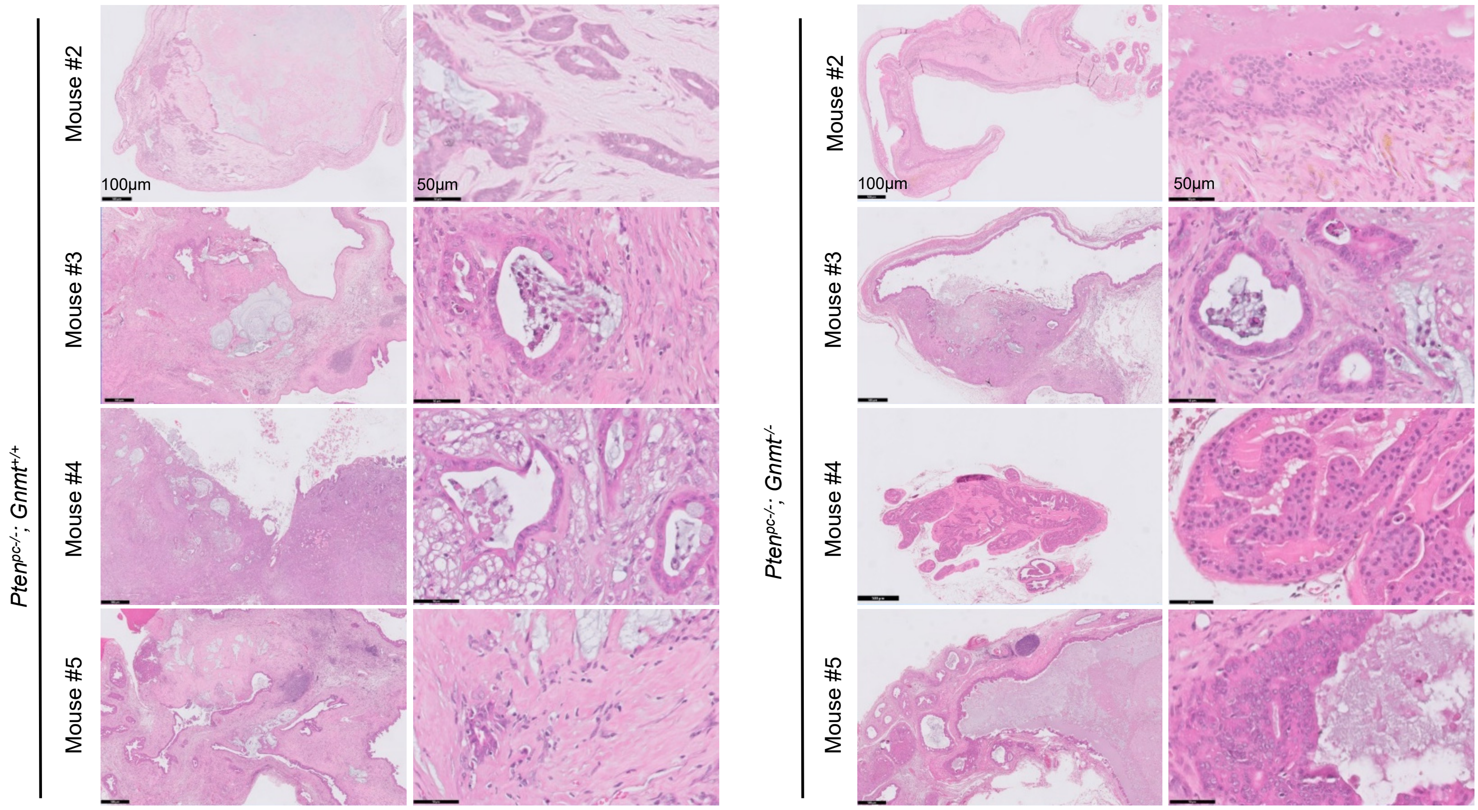

$Pten^{pc-/-}$; $Gnmt^{+/+}$
Mouse #2
Mouse #3
Mouse #4
Mouse #5
100µm
50µm
$Pten^{pc-/-}$; $Gnmt^{-/-}$
Mouse #2
Mouse #3
Mouse #4
Mouse #5
100µm
50µm

B

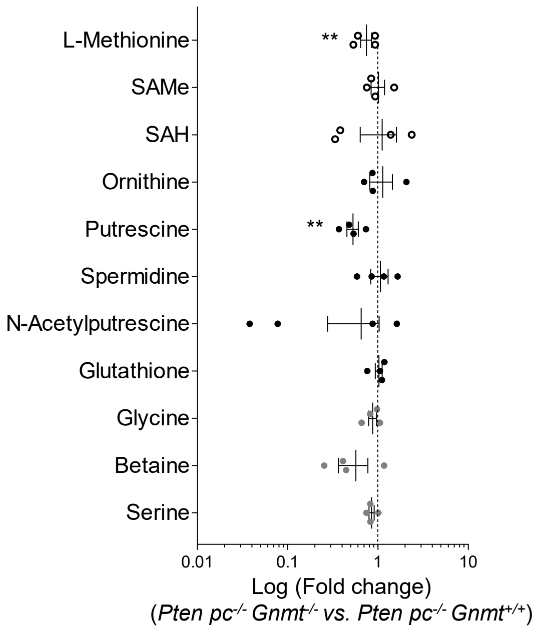

L-Methionine
SAMe
SAH
Ornithine
Putrescine
Spermidine
N-Acetylputrescine
Glutathione
Glycine
Betaine
Serine
**
**
0.01
0.1
1
10
Log (Fold change)
($Pten\ pc^{-/-}\ Gnmt^{-/-}$ vs. $Pten\ pc^{-/-}\ Gnmt^{+/+}$)

Figure S9

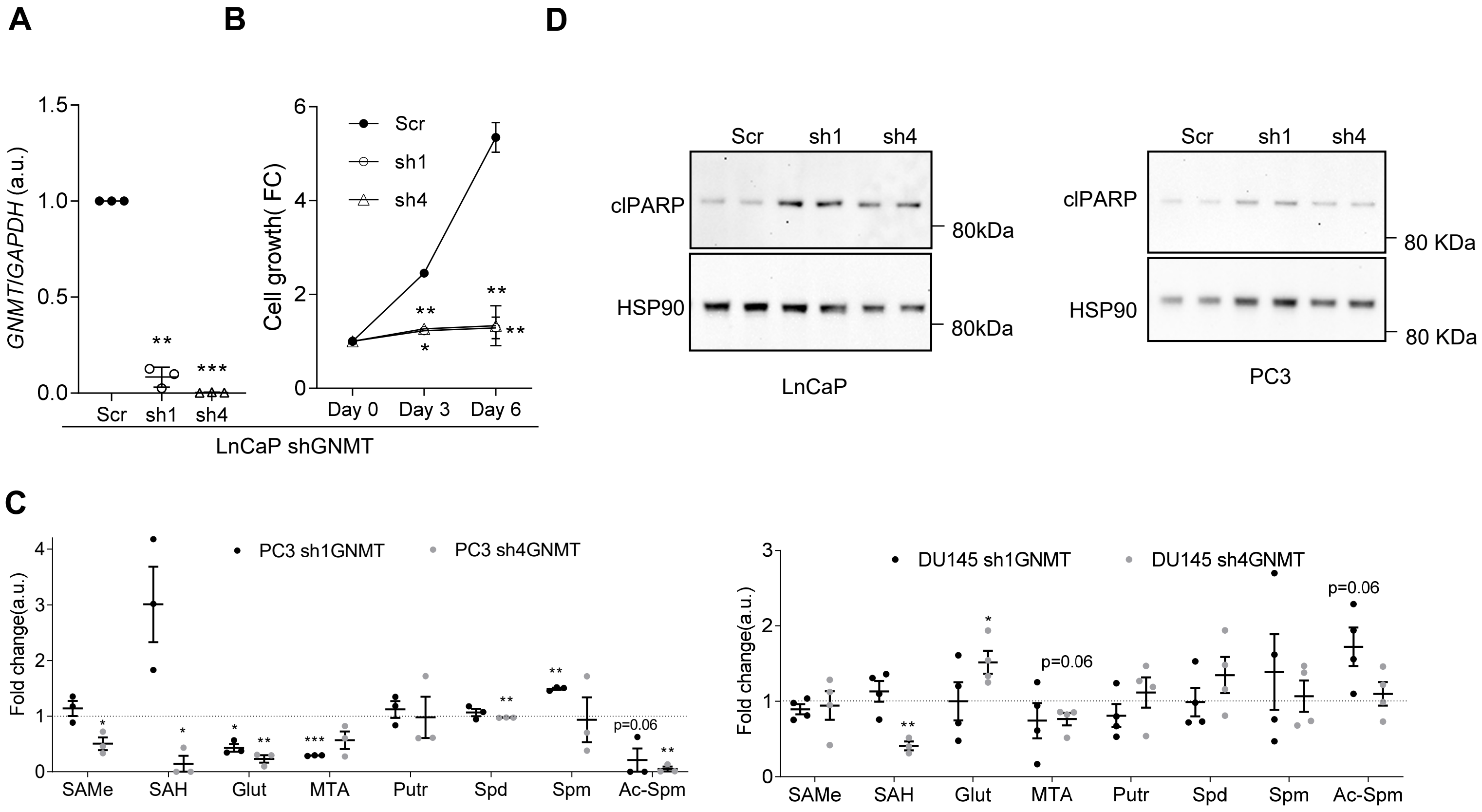
A
B
D
GNMT/GAPDH (a.u.)
1.5
1.0
0.5
0.0
**
***
Scr
sh1
sh4
Cell growth(FC)
6
4
2
0
Scr
sh1
sh4
**
*
**
**
Day 0
Day 3
Day 6
LnCaP shGNMT
Scr
sh1
sh4
clPARP
HSP90
80kDa
80kDa
LnCaP
Scr
sh1
sh4
clPARP
HSP90
80 KDa
80 KDa
PC3
C
Fold change(a.u.)
PC3 sh1GNMT
PC3 sh4GNMT
p=0.06
SAMe
SAH
Glut
MTA
Putr
Spd
Spm
Ac-Spm
Fold change(a.u.)
DU145 sh1GNMT
DU145 sh4GNMT
p=0.06
p=0.06
SAMe
SAH
Glut
MTA
Putr
Spd
Spm
Ac-Spm
